# Supplementary material for: Root Foraging Strategy Improves the Adaptability of Tea Plants (Camellia sinensis L.) to Soil Potassium Heterogeneity
Source: Int J Mol Sci. 2022 Aug 2;23(15):8585. doi: 10.3390/ijms23158585 (PMC9369073; doi:10.3390/ijms23158585)
Supplement: Supplementary file 1 [file ijms-23-08585-s001.zip › ijms-1823660-supplementary.pdf]

Supplementary Information for

Root Foraging Strategy Improves the Adaptability of Tea Plants (*Camellia sinensis* L.) to Soil Potassium Heterogeneity

Li Ruan [1,2], Hao Cheng [3], Uwe Ludewig [4], Jianwu Li [2,*] and Scott X. Chang [2,5,*]

1 Key Laboratory of Soil Contamination Bioremediation of Zhejiang Province, Zhejiang A & F University, Hangzhou 311300, China
2 Institute of Sericulture and Tea, Zhejiang Academy of Agricultural Sciences, Hangzhou 310021, China;
ruanl@zaas.ac.cn
3 National Center for Tea Improvement, Tea Research Institute, Chinese Academy of Agricultural Sciences, Hangzhou 310008, China; chenghao@tricaas.com
4 Institute of Crop Science, Nutritional Crop Physiology, University of Hohenheim, Fruwirthstr. 20, 70593 Stuttgart, Germany; u.ludewig@uni-hohenheim.de
5 Department of Renewable Resources, University of Alberta, Edmonton, AB T6G 2E3, Canada
* Correspondence: jameslee@zafu.edu.cn (J.L.); scott.chang@ales.ualberta.ca (S.X.C.)

Figure S1. A device for collecting root exudates from tea plant roots. An aerial view of the device for collecting root exudates **(A)**; Stereogram of the whole root exudate collection device **(B)**.

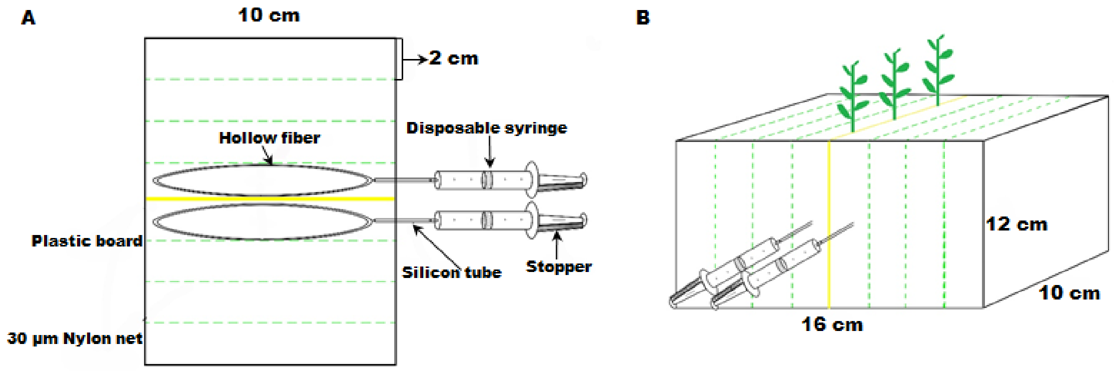

Table S1. Primers used in qRT-PCR experiments.

Gene ID	Forward 5'–3'	Reverse 5'–3'
TEA007938	GCTTTGGGGAGATGCTGA	GAGTTTTCCGACGAACGAC
TEA028696	ATCGTGAATCAGGTGGTATCT	GCATCCCTCAACAAAACAG
TEA006847	AGGTGTTGCTTCCAAAATG	TGGCGAATGAGTTCTGCTA
TEA010322	TAAACTATTACCCAAAATGCCC	AGAAAACCGCCCACGAAA
TEA034003	ATTACGGACATTGAGATACACTT	CACTCCATACTGAGAACCTTTG
TEA026325	GGATGGGATGTCAAGTATGC	TCTTCTGAGTGTTGCGAGTG
TEA021599	TTCCACGATTGCTTTGTTC	TGGCAGCCTCTACCACTTC
TEA019040	CGCACTATGACCCACTCTTC	TGTCCCTCAGTAAGTTCCATC
TEA008079	TTTGAACCCATCAGCGACT	CGAAACTCCTTGTCCACGT
TEA008459	CCCTACAGCCATAGTTACCG '	TGTTGTTTTCCACCTTAGCC
TEA030958	TCATCAACGCCAAGAACAC	GGTCGAGAACAGGGAGGG
TEA014414	TCTTGGTCGTCTTGCTTCC	CAGCCTTTATGTCTTCTCCAC
TEA012890	TTTCGTCAATGGGAGTGC	CAGGTTCCACCAACGCTAT
TEA025206	GGTTGGTCTTGGTTCAGGG	TCTAATGCGGGTTGCTCT
TEA023936	AATGGCTGTGACGGTTCTG	CATCTTGCCGCCCTACTG
TEA012804	TGGCATCTCGCCCGTAAA	CGCCCTTTCTCCCGCTCT
TEA012891	ATCTTCCCAGACCCACAAT	AGCACCACTCAAAGCCAC
TEA029495	TCCTCGTCCGACCACTCT	GGATTTTCACGGGTTCTTG
TEA023084	TGGAGGAGCAGGGACTAAA	TTGCTTATGCCACTGTTGAT
TEA005755	CTGTGAACAAAGCCATCTCC	CACGGCATAGTTTATCCCTC

Figure S2. Verification of the transcript levels obtained from RNA-seq using the qRT-PCR approach. Correlation analysis of the gene expression levels obtained from RNA-seq and qRT-PCR. Values are the log_2 ratio (treatment / control) of gene expression. The ratios (treatment / control) were as follows: Sp.SK / U.SK, Sp.NK / U.NK, and U.SK / U.NK. All qRT-PCR assays were performed with three biological replicates.

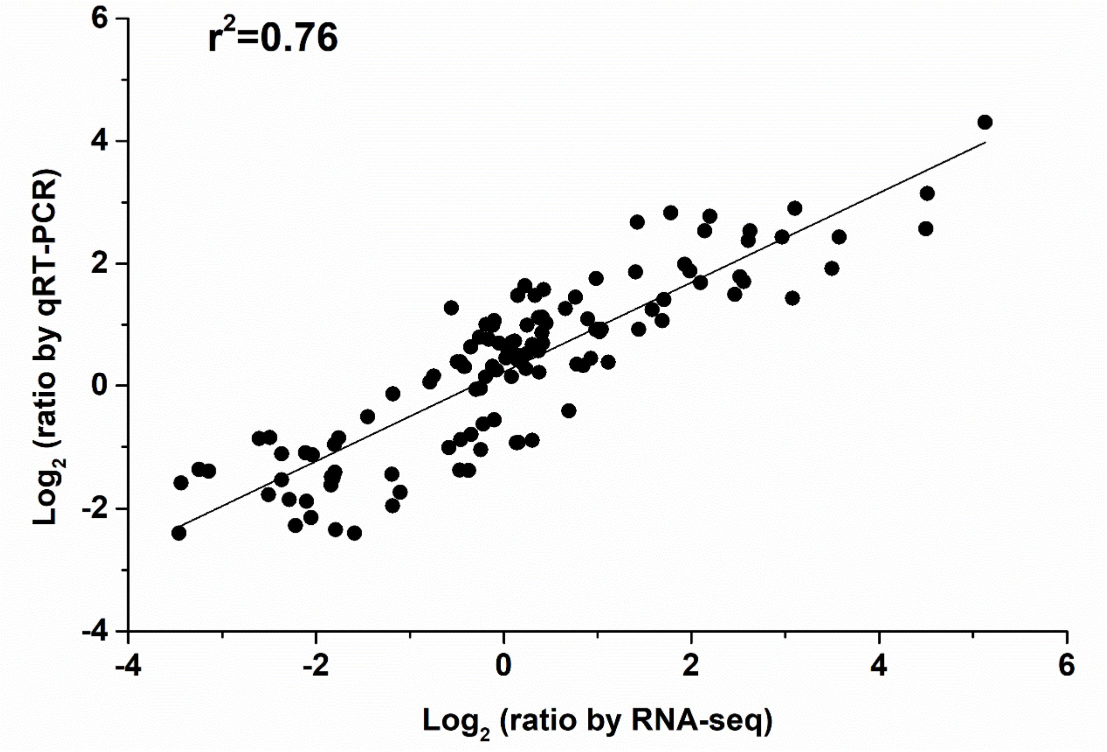

Figure S3. Temporal variations of organic acid contents of root exudates. Data are means ± SE (n=3).

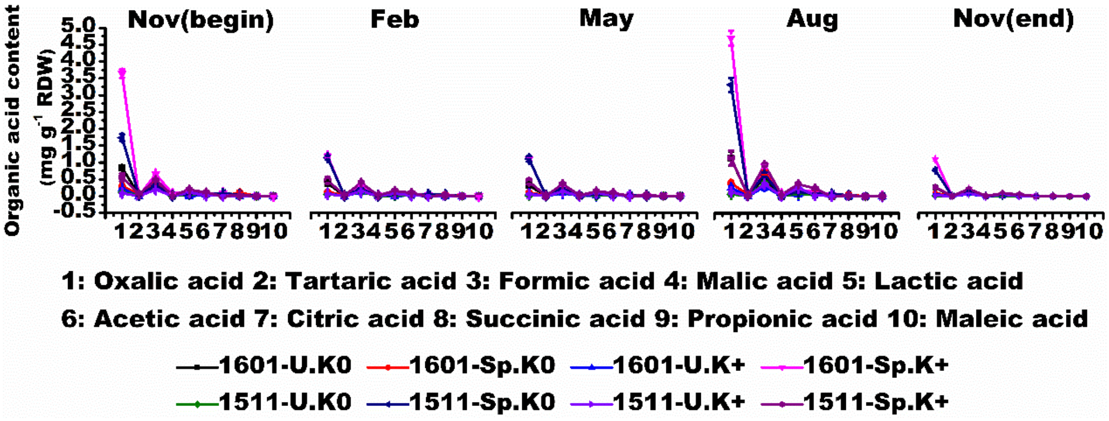

Figure S4. Relationship between cellulose decomposition gene expression and total root length.

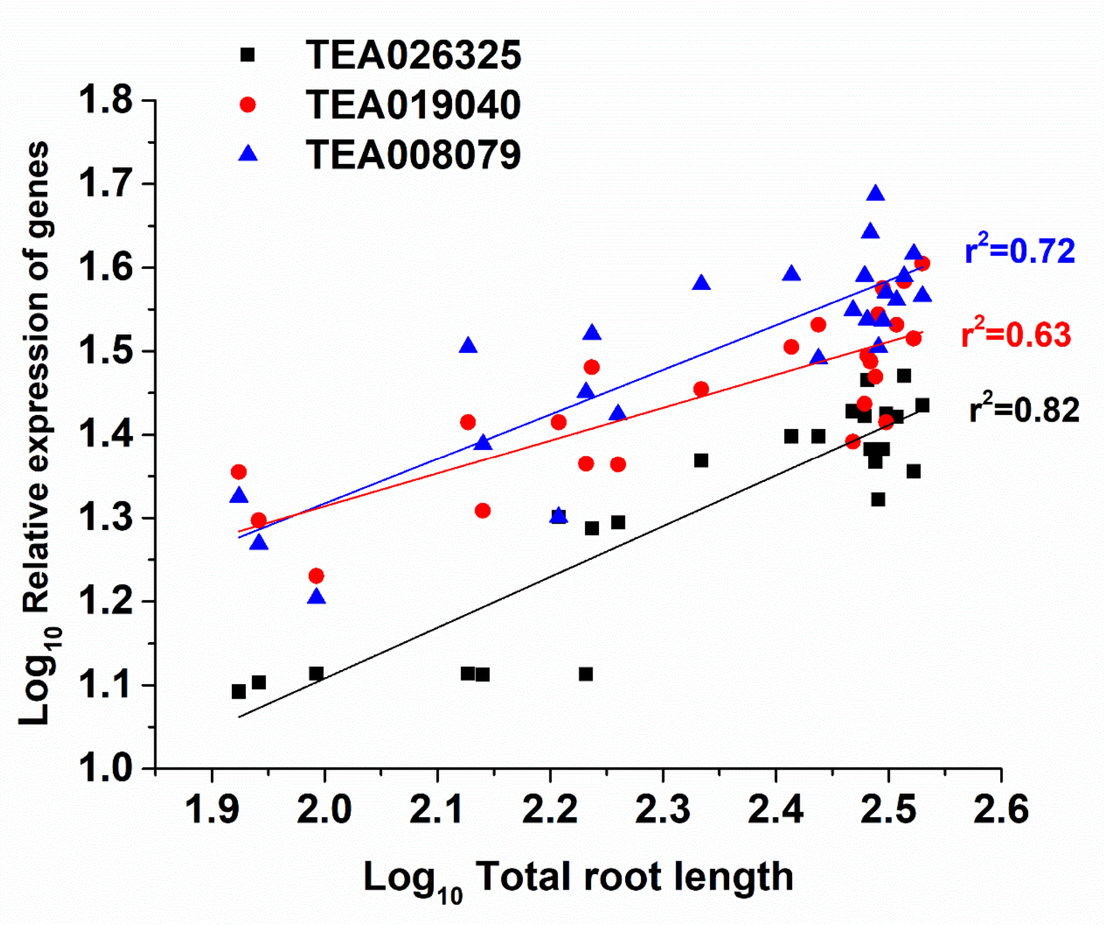

Figure S5. The activities of antioxidant enzymes and polyphenol contents in shoot （A）and root (B) systems of the two tea plant types when both were stressed by K limitation. Different letters represent significant differences at the level of $p < 0.05$.

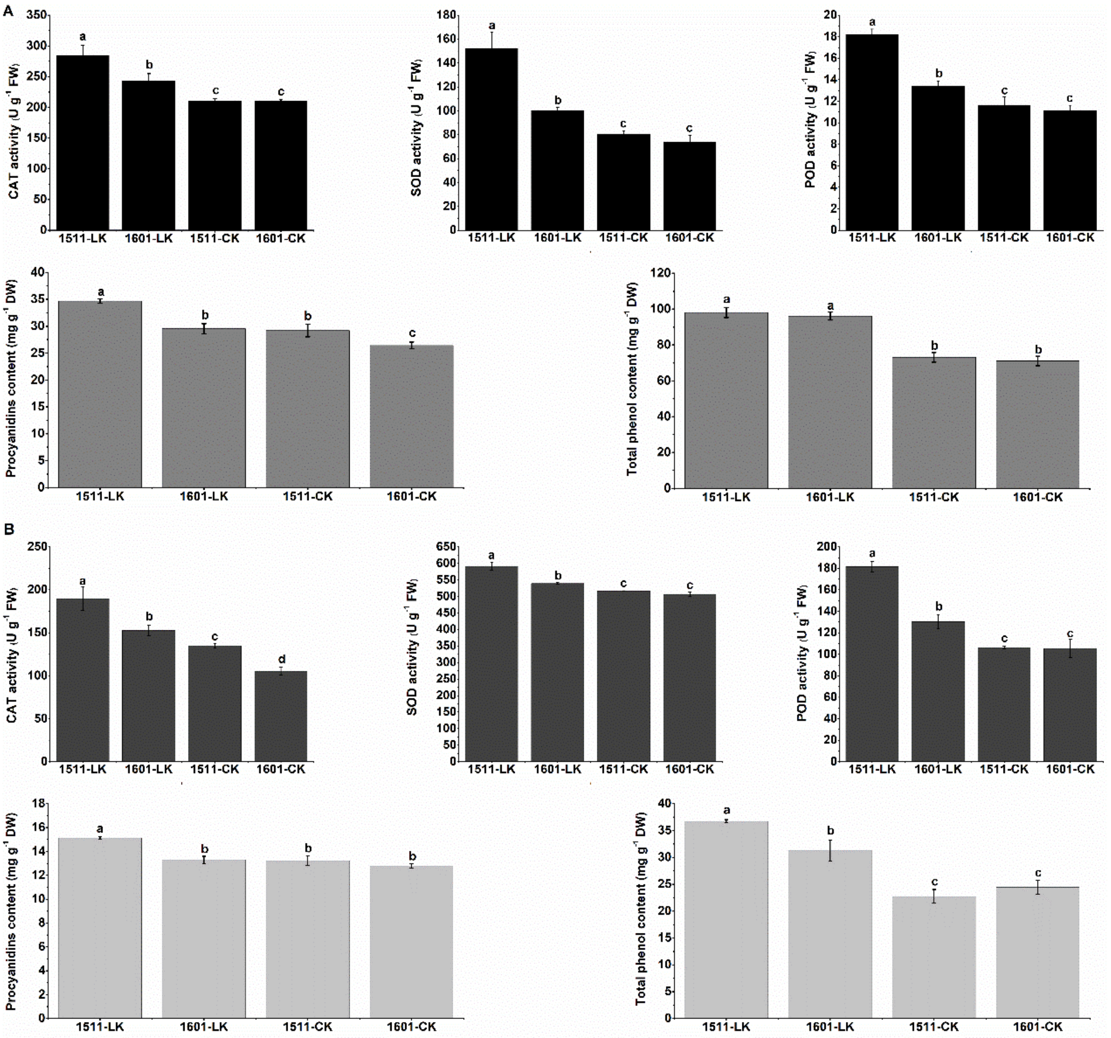

Gene ID	CHROM	POS	EFFECT
TEA003593	Scaffold2924	1841103	frameshift variant
TEA014837	Scaffold3721	530384	splice donor variant, intron variant
TEA013500	Scaffold980	117094	frameshift variant
TEA022207	Scaffold382	1916236	start lost
TEA024762	Scaffold7032	101455	frameshift variant
TEA010288	Scaffold2051	681239	frameshift variant
TEA010182	Scaffold13228	116725	frameshift variant
TEA023936	Scaffold9064	703151	stop gained
TEA013356	Scaffold6188	237037	frameshift variant, start lost

Table S2. Potential molecular markers of K tolerance for tea plants.
